# Rapidly progressing scalp melanoma in an elderly patient

**DOI:** 10.1002/ski2.431

**Published:** 2024-07-22

**Authors:** Christian Vestli, Assia Bassarova, Jose Hernán Alfonso

**Affiliations:** ^1^ Department of Dermatology Oslo University Hospital Rikshospitalet Oslo Norway; ^2^ Department of Pathology Oslo University Hospital Rikshospitalet Oslo Norway; ^3^ Department of Occupational Medicine and Epidemiology National Institute of Occupational Health Oslo Norway

## Abstract

A rapidly progressing scalp melanoma incorrectly diagnosed and managed as a ‘chronic wound’, lead to delayed referral to dermatologist and correct diagnosis. This case highlights the importance of early diagnosis of scalp melanoma and of frailty screening in elderly patients.

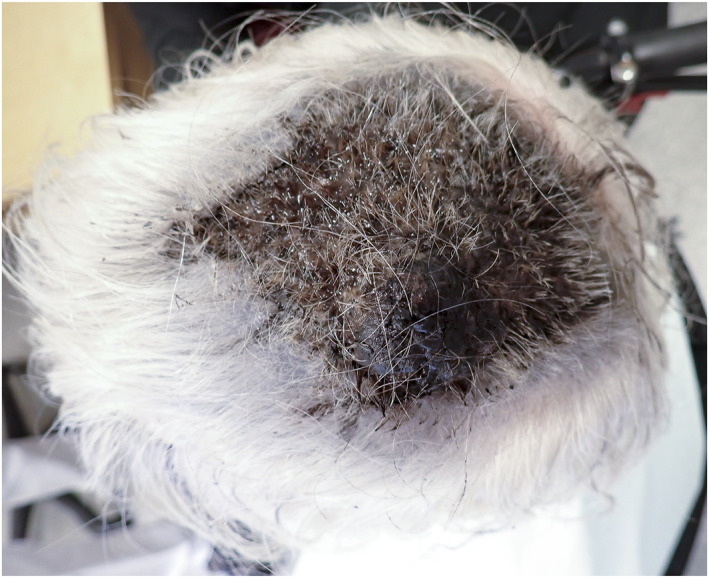

A woman in her 90's with multiple comorbidities, including advanced dementia was referred to the department of dermatology from a nursing home. She presented a massive scalp tumour (Figure 1a) and branched dark blue telangiectasia‐like lesions on the forehead (Figure 1b). The lesion was reportedly described as a small papulae and wound on the mid scalp 3 months prior to referral. We took a 5 mm punch biopsy from the left lateral forehead due to a high suspicion of malignancy. Histopathology confirmed scalp melanoma (Figure 1c, d). A palliative approach was chosen due to her comorbidities and the patient died shortly after due to advanced and untreatable melanoma.

**FIGURE 1:**
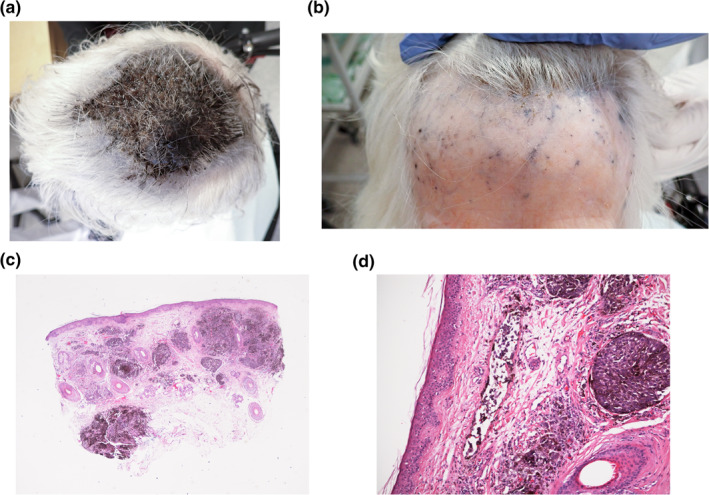
(a) Massive, painful, exophytic and protruding tumour (4 × 3 × 1, 5 cm) covering all the mid scalp over a thick black infiltrated plaque with necrotic areas (11 × 9 cm) from the frontal to the vertex of the scalp. (b) Dark blue telangiectasia with satellite lesions on the forehead and temporal regions representing local metastasis. (c) Diagnostic punch biopsy from forehead showing melanoma with irregular groups of hyperpigmented large atypical cells with vesicular nuclei. Breslow thickness was at least 3.1 mm, pTstage 3a, without ulceration and up to 4 mitosis per mm^2^. (d) Small tumour emboli in a superficial vessel.

Scalp melanoma behaves more aggressively compared to melanomas at other localisations, with higher recurrence and mortality rates.^1^ Early detection, timely surgical intervention, and customised treatment approaches are crucial to improve prognosis.^2^

Multiple comorbidities in elderly patients increase the risk of frailty leading to adverse health outcomes, institutionalisation and functional dependence.^3^ Therefore, frailty screening should be routinely included in elderly patients with rapidly progressing melanoma and other skin malignancies.^3^

## CONFLICT OF INTEREST STATEMENT

Jose Hernán Alfonso has received an unrestricted research grant from Sanofi. The other authors have no conflicts of interest to declare.

## AUTHOR CONTRIBUTION

**Christian Vestli**: Conceptualization (equal); funding acquisition (lead); investigation (lead); methodology (equal); project administration (supporting); visualization (supporting); writing–original draft (lead); writing–review & editing (supporting). **Assia Bassarova**: Visualization (equal); writing–review & editing (supporting). **Jose Hernán Alfonso**: Conceptualization (equal); funding acquisition (supporting); investigation (equal); methodology (equal); project administration (equal); resources (equal); supervision (lead); visualization (lead); writing–original draft (equal); writing–review & editing (lead).

## ETHICS STATEMENT

Not applicable.

## PATIENT CONSENT

The patient's family has provided written consent for usage of medical information and photographs to be published in print and online with an understanding that the information may be publicly available.

## Data Availability

Data sharing is not applicable to this article as no new data were created or analysed in this study.
